# Widening disparity in the geographic distribution of pediatricians in Japan

**DOI:** 10.1186/1478-4491-11-59

**Published:** 2013-11-22

**Authors:** Hiromasa Sasaki, Tetsuya Otsubo, Yuichi Imanaka

**Affiliations:** 1 Department of Healthcare Economics and Quality Management, Graduate School of Medicine, Kyoto University, Yoshida Konoe-cho, Sakyo-ku, Kyoto 606-8501, Japan

**Keywords:** Geographic distribution, Pediatrician, Physician, Workforce, Japan

## Abstract

**Background:**

The shortage of physicians in Japan is a serious concern, particularly in specialties like pediatrics. The purpose of this study was to investigate recent changes in the geographic distribution of pediatricians and the factors underlying this change.

**Methods:**

We investigated the numerical changes in the pediatrician workforce (2002 to 2007) per 100,000 of the population under the age of 15 years in 369 secondary medical areas throughout Japan, using attributive variables such as population size, social and economic status, and pediatric service delivery. We performed principal component analysis and multiple regression analysis.

**Results:**

We obtained two principal components: one that reflected the degree of urbanization and another that reflected the volume of pediatric service delivery. Only the first component score was positively correlated with an increased pediatrician workforce per 100,000 of the population under the age of 15 years. We classified the secondary medical areas into four groups using component scores. The increase in pediatrician workforce during this period was primarily absorbed into the two groups with higher levels of urbanization, whereas the two rural groups exhibited little increase. Pediatricians aged 50 to 59 years increased in all four groups, whereas pediatricians aged 30 to 39 years decreased in the two rural groups and increased in the two urban groups.

**Conclusions:**

The trends of the pediatrician workforce increase generally kept pace with urbanization, but were not associated with the original pediatrician workforce supply. The geographic distribution of pediatricians showed rapid concentration in urban areas. This trend was particularly pronounced among female pediatricians and those aged 30 to 39 years. Given that aging pediatricians in rural areas are not being replaced by younger doctors, these areas will likely face new crises when senior physicians retire.

## Background

The shortage of physicians is currently a serious social concern in Japan. The Democratic Party of Japan (DPJ) was elected to power in 2009, and committed to significantly increasing the number of medical students [1]. Scholars and administrative officials have discussed this problem at length and concluded that the main causes for the shortage of practicing physicians are the geographic maldistribution of physicians, increase in the proportion of female physicians, changes in physician distribution among specialties, and the introduction of a new training system for young physicians. These factors are addressed in further detail below.

The geographic maldistribution of physicians is not a new problem; instead, it is a persistent policy concern [2] that has long worried the government, and no fundamental solution currently exists [3]. Similar problems have been encountered in other countries [4-6].

The critical problem arising from the increase in the number of female physicians is that their career can ostensibly be negatively impacted by the birth of a child. In such cases, it is difficult for the physician to return to clinical practice if she is away for a prolonged period of time [7]. Several solutions for these problems have been implemented with some success [8-10].

Changes in physician distribution among specialties may also be related to the increase in female physicians, because the choice of specialty differs considerably between men and women. The physician shortage is particularly severe in pediatrics, obstetrics and gynecology, and anesthesiology [11,12], which are specialties that generally comprise a large proportion of female physicians.

The new system for training resident physicians commenced in the spring of 2004 [13]. Before the introduction of this system, most residents were trained in hospitals affiliated with medical schools. However, a large proportion of residents are now trained in town hospitals that have no direct association with medical schools [14]. Because hospitals affiliated with medical schools faced a substantial loss of their physician workforce, these hospitals began to poach physicians from town hospitals. This was possible because medical schools in Japan have considerable power over town hospitals with regard to personnel matters. The new system, which caused a shift in distribution and shortages of physicians in town hospitals within some regions, was intended to secure younger physicians to the workforce and train residents to become proficient in primary care. A town hospital may indeed be more suitable for primary care training than a medical college hospital.

Although several factors have been shown to contribute to the shortage of physicians, the most critical point to consider when intervening in the physician labor market is geographic distribution. In this study, we focused on recent changes in the geographic distribution of pediatricians, as pediatrics is one of the specialties in Japan that has a severe lack of physicians [15,16]. The aim of this study is to uncover the main determinants of variations in pediatrician distribution among geographic units.

## Methods

### Medical service administration areas

We used the secondary medical area (SMA) as the geographic unit of analysis. This is one of three levels of medical service administration areas legislated under the Medical Service Act. The primary level is the municipality, and the third and largest level is the prefecture. The second level lies between these two, and comprehensive primary care and emergency medicine are generally provided at this level. A total of 369 areas, designated as SMAs in 2005, were used in this study. SMA is possibly the most important of the three geographic classifications defined by the Medical Service Act with regard to implementing health care policies. The primary medical areas of municipalities and the tertiary medical areas of prefectures are based on local autonomy categories, and thus have little relevance or implications to the functionality of medical administration. On the other hand, the SMA has substantially more practical meaning as it refers to a geographic unit in which almost all medical service supplies are provided, except for the most advanced medical treatment. The SMA is also used as the basic unit for deciding the standard numbers of hospital beds within a region, which are mandated by the national government.

### Core workforce pediatricians

We analyzed the changes in the geographic distribution of pediatricians from 2002 to 2007. A pediatrician was defined as a physician whose primary specialty is pediatrics, as identified by physician profile data. We focused on pediatricians who were between 30 and 59 years of age during our targeted period (2002 to 2007), and refer to these pediatricians as ‘core workforce pediatricians’ (CWPs). Pediatricians were stratified by age, with categories of 30 to 39 years, 40 to 49 years, and 50 to 59 years. In Japan, physicians are classified as either hospital physicians or clinic physicians. Hospital physicians are employed by hospitals and treat both outpatients and inpatients. Clinic physicians are owners of clinics in which they exclusively practice, and only treat outpatients.

### Principal component analysis

We performed principal component analysis on the attributive variables of SMAs based on their correlations with one another. The attributive variables of SMAs were population size (2002), population density (2002), rate of population increase (2002 to 2006), population under 15 years of age (2002), population density under 15 years of age (2002), rate of population increase for those under 15 years of age (2002 to 2006), per capita income (2002), number of hospital beds per 1000 residents (2002), and CWPs per 100,000 of the population under 15 years of age (2002).

### Multiple regression analysis

Multiple linear regression analysis was used to identify factors associated with an increase in CWPs from 2002 to 2007, using the increase in CWPs per 100,000 of the population under 15 years as the dependent variable. We adopted the principal component scores determined by principal component analysis as explanatory variables in the multiple regression analysis. All analyses were performed using SPSS 15.0 J for Windows (SPSS Inc., Chicago, IL, USA), and statistical significance was set at *P* ≤0.05.

### Secondary Medical Area grouping

We classified SMAs based on whether principal component scores were positive or negative. Using the two principal components, we classified SMAs into four groups. We evaluated these groups by age (30s, 40s, or 50s), sex, and practice type (hospital or clinic) for pediatrician trends.

### Data sources

The number of pediatricians in each SMA was obtained from the physician database supplied by Nihon Ultmarc Inc. (Tokyo). This database comprehensively covers the statuses of physicians throughout Japan. Use of these data is permitted for medical science research and is monitored by an ethics committee composed of lawyers and scholars. The database includes each physician’s sex, birth year, specialty, practice style (that is, hospital or clinic), and the municipality where s/he practices.

Information regarding the population (2002 and 2006) and the population under 15 years of age (2002 and 2006) were determined from the Basic Resident Register Population published by the Ministry of Internal Affairs and Communication. Population density was calculated by dividing population by area of the region. Area of the region (2002) was obtained from the Geospatial Information Authority of Japan. Per capita income (2002) was obtained from the Ministry of Internal Affairs and Communication. The number of hospital beds (2002) was determined from statistics regarding medical facilities published by the Ministry of Health, Labour, and Welfare. Because we could not determine the number of beds for pediatrics, the number of hospital beds per 1000 residents was used as an alternative proxy variable.

## Results

Table 1 presents the descriptive statistics of attributive variables for the 369 SMAs. Table 2 presents the results of principal component analysis. For the first principal component, coefficients of the seven variables at the top of the table were large, whereas coefficients of the two variables at the bottom were small. The reverse was true for the second principal component. The choice between population and population density as an index for determining the degree of urbanization is usually not straightforward. In this study, however, the first principal component contained such a high proportion of both indices that we could confidently interpret this component as indicating the degree of urbanization. The coefficient for the rate of population increase was also large in the first principal component, indicating the concentration of population into urban areas. The first principal component also contained a high proportion of population indices for residents younger than 15 years of age, indicating few differences in the indices for the population of children and the total population of SMAs. Our findings that urbanized areas have higher per capita incomes were consistent with expectations. Given these considerations, the first principal component was interpreted as the degree of urbanization and level of demand for pediatric services. The second principal component was interpreted as the level of supply of pediatric services.

**Table 1:** Descriptive statistics (N = 369 secondary medical areas)

**Variable**	**Mean**	**SS**	**Minimum**	**Maximum**
Population	343797	383514	25070	2484326
Population density (/km^2^)	1056	2342	15	15774
Increase rate of population (%)	-1.20	2.57	-6.97	8.96
Population under 15 years old	49258	53444	3183	361868
Population density under 15 years old (/km^2^)	144	290	2	1729
Increase rate of population under 15 years old (%)	-6.30	4.51	-18.54	10.62
Income per capita (million yen)	1.24	0.30	0.65	2.99
Beds per 1000 population	10.51	3.47	1.43	24.90
CWP per 100,000 population under 15 years old	50.19	30.93	0	393
Increase of CWP per 100,000 population under 15 years old	2.01	16.63	-123.11	61.46

CWP, core workforce pediatricians.

**Table 2:** Principal component analysis results (after varimax rotation)

	**1st principal component**	**2nd principal component**
Population	0.859	0.013
Population density	0.844	0.050
Increase rate of population	0.798	0.017
Population under 15 years old	0.838	-0.018
Population density under 15 years old	0.868	0.001
Increase rate of population under 15 years old	0.847	0.024
Income per capita	0.865	0.042
Beds per 1000	-0.250	0.847
CWP per 100,000 of the population under 15 years old	0.316	0.818

The results of the multiple regression analysis are presented in Table 3. The first principal component score showed a significant positive correlation with the dependent variable (increase in CWPs per 100,000 population under 15 years), whereas the second principle component did not. This result indicates that more urbanized SMAs have more CWPs. The demand for pediatric services strongly correlated with CWP increase, whereas the supply of pediatric services showed little correlation with quantitative changes in CWPs.

**Table 3:** Multiple regression analysis results

**Explanatory variable**^**a**^	**β coefficient**	**Standard error**	***P *** **value**
Constant	3.580	0.849	<0.001
1st principal component score	5.328	0.850	<0.001
2nd principal component score	0.012	0.850	0.895

R^2^ = 0.097.

^a^Dependent variable is the increase of core workforce pediatricians (CWP) per 100,000 of the population under 15 years of age.

**Figure 1:**
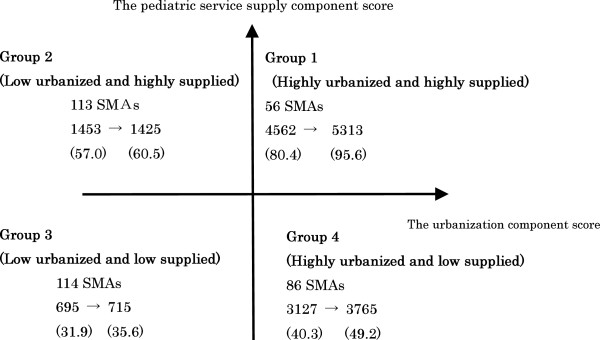
The groups of secondary medical areas (SMAs) and changes in the number of core workforce pediatricians.

Using the two principal components, we divided SMAs into four groups based on whether component scores were positive or negative (Figure 1, Table 4). Figure 1 shows the changes in CWPs of each group from 2002 to 2007. Groups 1 and 4 experienced the largest increases in CWPs. In Groups 2 and 3, CWPs leveled off. The total increase in CWPs during this period was therefore primarily absorbed into the urban areas of Groups 1 and 4. There was no group that demonstrated a decrease in the pediatric workforce per 100,000 of the population under 15 years old.

**Table 4:** The means of each group’s variables

	**CWP (2002)**	**CWP (2007)**	**Population (2002)**	**Population under 15 (2002)**
Group 1	81.5	94.9	720,750	101,330
Group 2	12.9	12.6	160,412	22,561
Group 3	6.1	6.3	133,410	19,274
Group 4	36.4	43.8	618,184	90,175

CWP, core workforce pediatricians.

The features of the four SMA groups were as follows: Group 1 was urbanized and abundant in pediatric services in 2002. This group experienced a large increase in CWPs from 2002 to 2007. Most SMAs in this group contain one or more university hospitals, which gives them an advantage in procuring a pediatrician workforce. Group 2 was relatively rural, but had abundant pediatric services in 2002. SMAs in this group have large municipality hospitals, and some contain university hospitals. CWPs of this group decreased slightly from 2002 to 2007. The large decreases in pediatricians in their 30s or 40s and those employed by hospitals are noteworthy. The decrease in younger members of the workforce would effectively result in a de facto pediatrician shortage in the SMAs of this group, because younger pediatricians usually perform more laborious work such as night shifts and emergency medicine. Group 3 was rural, and pediatric services were scarce. This group experienced no increase in CWPs, and a decrease in the younger workforce was observed. The situation exhibited in this group is consistent with the commonly held idea that rural areas tend to have a shortage of physicians. Group 4 was urbanized and lacked pediatric services, but experienced a large increase in CWPs from 2002 to 2007. The younger workforce also increased in SMAs of this group. Many SMAs in this group are located in the suburbs of big cities.

**Figure 2:**
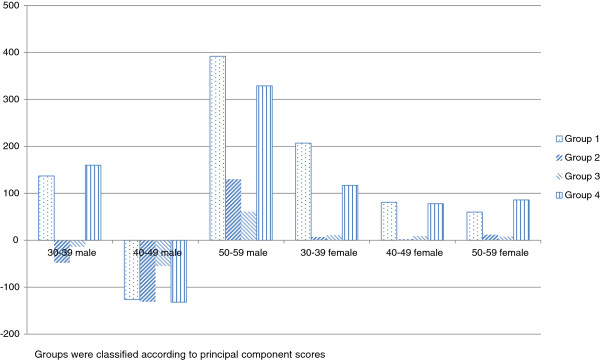
Core workforce pediatrician increases by sub-categories of age and sex for each group.

As shown in Table 5, CWPs increased during this period and were consistent with the changes in numbers of physicians of all types. Table 5 and Figure 2 show changes in pediatrician numbers for each group by sex, age group, and practice type. The gap in the rate of pediatrician increase between urban and rural areas was larger in the 30 to 39 year age group than in more senior age groups. Because most young pediatricians are hospital physicians, the increase in hospital pediatricians in Groups 1 and 4 was considerably larger than the increase in clinic pediatricians. The increase rates of female pediatricians were larger than those of their male counterparts in all categories under 50 years of age. In particular, large increase rates were observed in females in their 30s in Groups 1 (+41%) and 4 (+39%).

**Table 5:** The numbers of pediatricians in 2002 and 2007 by group (Group 1 to 4), age group, practice type, and numbers of all types of physicians

**Group**	**Age group**				**Practice type**			
		2002	2007	Increase rate %		2002	2007	Increase
		(female)	(female)	(female)				rate %
Pediatricians								
Group 1	30-39	1517 (509)^a^	1861 (716)^a^	+23 (+41)^a^	Hospital	3457	4104	+19
	40-49	1768 (397)	1723 (478)	-3 (+20)	Clinic	1105	1209	+9
	50-59	1277 (300)	1729 (360)	+35 (+20)				
	30-59	4562 (1206)	5313 (1554)	+16 (+29)				
Group 2	30-39	443 (136)	402 (143)	-9 (+5)	Hospital	1026	998	-3
	40-49	597 (104)	468 (106)	-22 (+2)	Clinic	427	427	0
	50-59	413 (85)	555 (97)	+34 (+14)				
	30-59	1453 (325)	1425 (346)	-2 (+6)				
Group 3	30-39	193 (40)	190 (51)	-2 (+28)	Hospital	470	486	+3
	40-49	285 (48)	239 (57)	-16 (+19)	Clinic	225	229	+2
	50-59	217 (44)	286 (52)	+32 (+18)				
	30-59	695 (132)	715 (160)	-3 (+21)				
Group 4	30-39	814 (300)	1091 (417)	+34 (+39)	Hospital	2016	2559	+27
	40-49	1333 (294)	1279 (372)	-4 (+27)	Clinic	1111	1206	+9
	50-59	980 (217)	1395 (303)	+42 (+40)				
	30-59	3127 (811)	3765 (1092)	+20 (+35)				
Total	30-39	2967 (985)	3544 (1327)	+19 (+35)	Hospital	6969	8147	+17
	40-49	3983 (843)	3709 (1013)	-7 (+20)	Clinic	2868	3071	+7
	50-59	2887 (646)	3965 (812)	+37 (+26)				
	30-59	9837 (2474)	11218 (3152)	+14 (+27)				
All types of physicians								
Total	30-39	59,970 (10,864)	60,961 (14,734)	+2 (+36)				
	40-49	65,256 (7107)	70,563 (10,234)	+8 (+44)				
	50-59	41,135 (3776)	57,887 (5786)	+41 (+53)				
	30-59	166,361(21,747)	189,411 (30,754)	+14 (+41)				

^a^The numbers shown in parentheses indicate the number of females in each category.

## Discussion

From the attributes of geographic units (that is, SMAs), we obtained two independent principal components: the degree of urbanization and the level of pediatric service supply. The degree of urbanization and the level of pediatric service supply were determined to be independent of each other. This discovery is interesting in that it contradicts the conventional idea that the volume of medical service supply tends to be larger in urban areas than in rural areas. The situation in areas like Group 2 (low urbanization and high supply) suggests that rural areas can also be rich in medical resources. On the other hand, our findings show that urban areas like Group 4 (high urbanization and low supply) were not always successful in acquiring an adequate pediatrician workforce. We therefore surmise that before the study period, social policy was relatively successful in distributing pediatricians equally between rural and urban areas. The trend of physician concentration in urban areas is persistent, but social policies are sometimes able to resist this trend.

We will discuss in this paragraph the ‘mean’ we referred to above for the social policies. In the past, prefectural governments and ‘*ikyoku*’ systems worked together to allocate pediatricians to rural areas [17-19]. Prefectural governments create programs aimed at distributing medical resources appropriately and equitably. Yet, governments must request cooperation from many other entities to successfully implement these programs. The *ikyoku* system is one such entity, and plays a critical role in the training and allocation of physicians. *Ikyoku* can be thought of as a temporary staffing company for physicians in that it is congruent with prefectural government policy aimed at distributing medical resources equally. Under this system, physicians are assigned to clinics and hospitals in rural areas, which are often unpopular as work and living destinations for physicians. *Ikyoku* is a term for the organizational theory of Japanese medical care. The word originally refers to a ‘staff room for physicians,’ but now has the broader connotation of ‘physicians’ guild.’ *Ikyoku* is a hierarchical system with a professor of clinical medicine as its head, who is frequently the chief of a department covering a particular specialty (for example, cardiovascular medicine, pediatrics, or gastrointestinal surgery). Member physicians in an *ikyoku* system are rotated and promoted among hospital positions within the system. Most of these are large teaching hospitals located in both urban and rural areas. Thus, *ikyoku* systems could play monopolistic roles in training physicians and allocating them to hospitals, while taking urban–rural equity into consideration.

From 2002 to 2007, the majority of the increase in CWPs was absorbed into the SMAs of Groups 1 and 4, which are urbanized areas. The trend of pediatrician concentration in urban areas was particularly evident among female physicians and those in their 30s. In Groups 2 and 3, which are rural areas, CWPs leveled off and an aging of the pediatrician population was observed. The number of pediatricians in their 50s increased, whereas the number in their 30s and 40s decreased, indicating that few younger pediatricians entered the workforce in these areas. This implies that the shortage of pediatricians in rural areas will become more serious in the near future when senior pediatricians retire.

This large shift in pediatrician distribution can be partly attributed to the recent decline in the power of *ikyoku* systems [19,20]. The new system for training resident physicians, which was implemented in 2004, has created a kind of free labor market. Young physicians are now able to manage their own career paths by applying for resident or senior positions in hospitals. *Ikyoku* systems were designed to promote medical studies, produce expert physicians, and allocate the physician workforce to various areas. However, young physicians tend to be more interested in developing their skills, and are less interested in medical studies or obtaining degrees. Therefore, the *ikyoku* system has fallen out of favor as a career path for many young physicians. Alternative systems for the *ikyoku* system should be implemented to promote physician mobilization between urban and rural areas.

Social policies that increase the number of rural physicians can be classified into six categories based on timing and target (Table 6). Regulations require a physician to work in rural areas by legal force or custom. Although entering the *ikyoku* system is optional, members of this system do not have a choice regarding where they practice. We therefore classified this system into the regulation category. The preference for living in urban areas is attributed to the desire for the latest medical information and availability of career opportunities. Physicians must also consider educational opportunities for their children and preferences of their spouses [2]. Work-life balance issues are therefore part of these considerations.

**Table 6:** Classification of the measures to increase the number of physicians in rural areas

**Target**	**Regulatory**	**Financial incentive**	**Work-life balance**
**Timing**			
Before practice	・Special entrance examinations for high school students of local origin	・Conditional scholarships with stipulations on future rural practice	・Exposing medical students to rural practice
After practice	・*Ikyoku* system	・High salaries for rural physicians	・Supporting rural physicians for both life and practice
	・Restriction to location		

The policies adopted so far in Japan are primarily in the financial incentive or regulation categories, and fewer policies have targeted work-life balance. However, financial incentives have unclear effects [21], and regulation policies also present hurdles. Thus, future policies must combine these three categories, and a shift toward work-life balance policies may be desirable.

This study has some limitations. First, pediatricians in their 20s or 60s were excluded from this study, which may possibly introduce sample bias. Although some pediatricians in their 60s contribute substantially to primary care in their communities, there appears to be a large variation in how they practice compared with younger pediatricians. Under the new system for training resident physicians, residents must practice as general physicians without specialization. This led to a decline in the number of pediatricians in their 20s during the study period (2002 to 2007), as the new system for training resident physicians was initiated in 2004. Accordingly, we limited the pediatrician age group to 30 to 59 years. Second, although we focused only on pediatricians, physician labor market-associated phenomena (for example, an increase in female practitioners in the physician workforce , shortage of physicians in rural areas, change in the power of *ikyoku* systems) are not unique to pediatrics. Thus, future studies should be extended to include physicians of other specialties. Third, this study is retrospective and does not provide conclusive evidence regarding transcending times. In social sciences, collecting descriptions on limited facts sometimes results in breakthroughs, and historical studies such as this therefore have value in publication. Fourth, there may be several municipalities where the numbers of pediatricians are low but have an efficient system to transfer children requiring tertiary care to a larger hospital. However, the SMA is defined as the geographical unit in which the delivery of comprehensive primary care is possible, and the transportation factor is probably more pertinent at the level of a larger geographical unit, such as tertiary medical areas. At the level of SMAs, we believe that the omission of this transportation factor would have a negligible impact on the results.

## Conclusions

Our study revealed that the geographic distribution of pediatricians substantially changed between 2002 and 2007. Underlying this change was a weakening of systems that allocate physicians to rural areas, leading to a concentration of pediatricians in urban areas. Notably, this trend was particularly pronounced among female physicians and those in their 30s. Given that pediatricians in rural areas are aging, these areas will face new crises in the near future when senior pediatricians retire. We propose a policy shift from financial incentives and regulations to measures that emphasize work-life balance in order to promote a better urban–rural distribution of pediatricians.

## Abbreviations

CWP: Core workforce pediatricians; SMA: Secondary medical area.

## Competing interests

All authors declare that they have no competing interests.

## Authors’ contributions

All authors participated in the study design. HS carried out the analyses and drafted the manuscript. TO collected data and supervised the data analysis. YI supervised the total study process and helped to draft the manuscript. All authors read and approved the final manuscript.
